# Seasonal dynamics and adaptation strategies of krummholz forming Rhododendron campanulatum to water availability at high-altitude Himalayan treeline environments

**DOI:** 10.1371/journal.pone.0318197

**Published:** 2025-02-24

**Authors:** Nandan Singh, Ashish Tewari, Shruti Shah, Amit Mittal, Saurabh Gangola, Zishan Ahmad Wani

**Affiliations:** 1 Department of Forestry and Environmental Science, Kumaun University, Nainital, Uttarakhand, India; 2 Faculty of Agriculture and Agroforestry, Kumaun University, Nainital, Uttarakhand, India; 3 School of Allied Sciences, Graphic Era Hill University, Nainital, Uttarakhand, India; 4 Department of Microbiology, Graphic Era deemed to be University, Dehradun, Uttarakhand, India; 5 GB Pant National Institute of Himalayan Environment, Centre for Biodiversity and Conservation Management, Kosi-Katarmal, Almora, Uttarakhand, India; Central South University of Forestry and Technology, CHINA

## Abstract

This study investigates the water relations mechanism of *Rhododendron campanulatum* (D. Don) in treeline areas of the Himalayan region, emphasizing its vital role in ecosystem dynamics. The species, commonly found as krummholz forms at treeline ecotones, exhibits notable population advancement 1.4 m/yr, which could lead to the densification of the ecotone in the future. Seasonal measurements of soil moisture at depths of 15cm, 30cm, and 45cm, as well as pre-dawn (Ψ_PD_) and mid-day (Ψ_MD_) water potentials, and leaf conductance in the forenoon (gw_AM_) and afternoon (gw_PM_), were conducted for both trees and seedlings. Significant variations were observed among sites, seasons, and years across various parameters, including soil moisture and water potential components. The study underscores the most negative water potentials (Ψ) during winters, with Ψ_PD_ reaching -0.88 MPa for trees and -1.43 MPa for seedlings. Seasonal changes in water potential (ΔΨ) ranged from 0.11 to 0.82 MPa for trees and 0.20 to 0.90 MPa for seedlings. Osmotic potential at full turgor (OP_Full_) declined from winter to summer, ranging from -0.74 to -1.76 MPa in trees and -0.45 to -1.60 MPa in seedlings. Relative water content (RWC%) of seedlings varied between 68.00% and 87.40%. Leaf conductance ranged from 19.50 to 329.68 m mol m^-2^ sec^-1^ in trees and 43.60 to 331.40 m mol m^-2^ sec^-1^ in seedlings. The study highlights the adaptation of *R*. *campanulatum* and other broad-leaved evergreen species to high-altitude climatic irregularities, emphasizing the crucial role of snowmelt and monsoon rains in mitigating water stress. Osmotic adjustment and high relative water content enable *R*. *campanulatum* to cope with environmental changes, maintaining water availability for photosynthesis.

## Introduction

*Rhododendrons*, belonging to the Ericaceae family, are a diverse genus of woody plants primarily found in the Northern Hemisphere, ranging from North America across Europe and Asia to Japan, extending from the extreme north to the Equator [1]. In the Himalayas, *Rhododendrons* play a vital role in the ecosystem and are commonly found as small trees, shrubs, or krummholz forms at treeline ecotones, at an elevational range of 3000-4200m [2]. Treeline species thrive in humid temperate regions with highly organic, well-drained acidic soils, and they have adapted to the challenging climate categorized by dry, calm, and cool summer season, and extended severe winter season [3]. However, a warming climate is impacting *Rhododendron* populations in the Himalayan region [4,5]. The rate of upward advancement of *R*. *campanulatum* population was computed at 1.4 m/yr in the elevation transect of the Tungnath site (3511–3665 m a msl) [4]. The upward migration due to climate change may alter habitat structure and influence species interactions, potentially leading to shifts in local biodiversity [6]. Understanding these consequences is crucial, as they can affect overall ecosystem resilience and functionality [7]. The interspaces between the krummholz patches were observed to be filling up with regenerating individuals [8], which could lead to the densification of the ecotone in the future. However, despite decades of warming, the treeline did not show any significant upward movement [4]. *R*. *campanulatum* plays an important role in soil stabilization, thus prevent erosion in the fragile Himalayan ecosystems, and influences local water cycles through transpiration and water retention, contributing to the overall health of the ecosystem [9]. These services emphasize the importance of preserving *R*. *campanulatum* in the context of global warming.

The areas of tall mountainous are affected by multifaceted connections between ecological courses and environmental aspects [10]. In current years, global climate change has resulted in increasing temperatures and decreasing precipitation [11], resulting in drought occurrences worldwide [12], that can severely impact natural ecosystems, particularly in treeline areas of the Northern Hemisphere especially during winter, leading to tree seedling mortality [13]. The shift from snow to rain may increase drought stress in the growth season for trees [14]. The change ability of microsites plays a crucial part in the relationship between subsistence and drought with the altering climate. In the alpine environments, winter conditions can impair plant-soil water relations, influencing the life of a tree in the subsequent summer season [15]. In monsoonal climates trees also face significant water deficiencies, and produce diverse responses and adaptations to drought for species are formed at different times of the year [13,16]. Winters with snow cover result in soil freezing and water uptake blockage, leading to lethal water deficits in treeline ecotone areas [17], especially affecting young tree individuals (seedlings) at drier sites [18]. Severe drought events can also significantly impact natural ecosystems [13], and with climate change, there could be a shift from snow to rain, earlier depletion of snow cover, and increased evapotranspiration, leading to drought conditions for plants [13].

The movement of water from the soil through plants to the atmosphere occurs along a gradient of water potential [19], with the lowest Ψ typically found at the leaf surface. The water potential of trees and their various components play a vital role in the physiology and metabolism of plants [19]. Ψ_PD_ varies in response to environmental gradients [20], indicating how plants integrate soil water availability. As a result, Ψ_PD_ serves as a valuable measure of plant water status and may be correlated with maximum stomatal conductance [21,22]. Understanding the mechanisms that govern plant Ψ_PD_ is essential for realistically interpreting plant water use, understanding plant adaptation to stress, and evaluating the water balance of plant communities [23,24]. To withstand water supply for leaf expansion, plants may adjust osmotic potential and tissue elasticity [25], and condensed osmotic potential permits plants to sustain turgor and cell enlargement even at low water potentials [21,26]. Drought can lead to the regulation of transpiration in leaf through stomata; and damage of hydraulic conduction in twigs [27]. Transpiration is determined by the dampness between the atmosphere and the leaves with temperature effects during winter influencing increased transpiration due to overheating [28,29].

*Rhododendrons* in the Indian Himalayan treeline region have been less studied, particularly in terms of the impact of drought and its consequences on tree mechanism [4,13,21], as well as their regulatory role in the distribution and performance of species [30]. Trees growing in treeline areas face multiple environmental stresses that influence their water relations [31,32]. Leaf water status is closely related to various leaf physiological variables, including turgor, growth, stomatal conductance, transpiration, photosynthesis, and respiration [33]. During the growing season in treeline species, leaf conductance is generally not restricted, allowing more open stomata and higher conductance, even when leaf water potential decreases, indicating potentially higher photosynthesis and transpiration rates [34]. Trees may adjust osmotic potential control to reduce water loss or maintain xylem conductance to ensure water supply for leaf enlargement [25]. Osmotic adjustment is an effective adaptation used by tree species to overcome drought, as it helps maintain turgor during drought periods and enhances the competitiveness of species in treeline areas [13,35]. Studies have shown that treeline species worldwide exhibit similar physiological adaptations to changing climates, such as shifts in phenology and increased stress tolerance [36]. In the present study, we sought to understand the mechanisms of tree water relations in *R*. *campanulatum* at treeline areas in the western Himalayan region. Our research endeavors to provide crucial insights into how these trees navigate water stress, adapt to dynamic climatic conditions, and influence the functioning of Himalayan ecosystems. This study bridges gaps in the existing literature by addressing the specific challenges and adaptations of *R*. *campanulatum* within the unique context of the western Himalayas, thus offering valuable contributions to the scientific discourse on mountain ecology and climate resilience.

## Materials and methods

### Sites of the study

**Fig 1:**
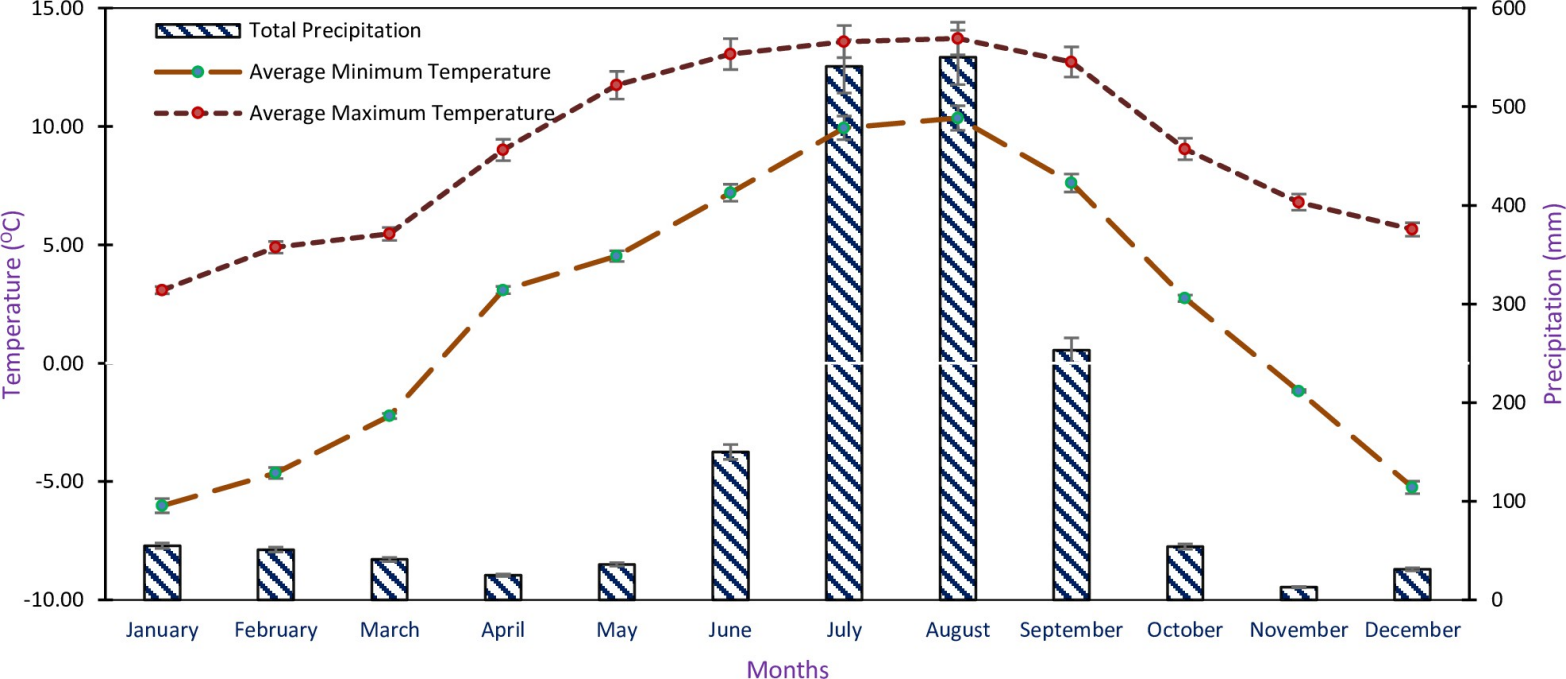
The mean monthly data of temperature and precipitation at Tungnath study site during 2017–2020 (Source: IHTP).

The research was carried out at three treeline sites: Tungnath, Bedni, and Aali, situated in the western Indian Himalayan region at coordinates 30°11´02˝N and 79°39´36˝E. The rationale behind selecting the specific study site, because these sites are positioned at elevations ranging from 3145 to 3467 m and fall within the alpine and the sub-alpine zones based on their representation of a diverse altitudinal gradient and unique microclimatic conditions. These locations feature varied elevation, temperature, precipitation, and crucial for studying the climatic effects on *R*. *campanulatum’s* physiological responses. There were sporadic studies on these sites especially with reference to the tree water relations of *R*. *campanulatum* in the Indian Himalayan treeline environment. These areas comprise brown-colored soil, with a sandy loam texture containing a higher proportion of sand and silt. Additionally, the pH values of the soil range between 4.0 to 5.0 which portrays that the soil tends to be acidic [22]. The monsoon affects the climate of the area of the study and is characterized by long severe winters and short cool summers. The mean monthly minimum and maximum temperature varied between -6.02±0.23 to 10.36±0.43°C, and 3.08±0.76 to 13.71±1.03°C and the total monthly precipitation was 13.0±1.16 and 541.0±4.37 mm during 2017–2020 at Tungnath treeline site [37] (Fig 1).

### Measurements

The study was carried out at three designated sites; at each site, a 1.0-hectare area was selected and all measurements were conducted within this marked area. At these marked areas, 10 representative phenotypically superior individuals of varying circumference ranging from 33 to 50 cm were randomly selected for measurement. To assess tree and seedling density, 10×10 m measuring quadrats were used for the trees and 1×1 m for the seedlings. Criteria were followed to distinguish between trees and/or seedlings [38]. For evaluating soil moisture (Sm), measurements were taken at three different soil depths. Additionally, leaf conductance (gw), water potential (Ψ) of tree twigs, and components of water potential were measured on illustrative trees and seedlings. The study was conducted between 2017 to 2020, with a total of 48 samplings taken at seasonal intervals. These intervals were categorized between March and May as the summer season; the period between June and September was categorized as the rainy season; the period between October and November autumn season; and between December and February as winter. This seasonal approach allowed us to observe the disparity in the parameters throughout the duration of the study.

### Moisture in soil

For the estimation of soil moisture, the soil samples were collected from five different locations at three different levels of soil depths: 0–15 cm, 15–30 cm, and 30–45 cm, for better understanding of the interaction between soil, water, and plants, for water relations and ecosystem dynamics studies. Each study site used a stainless-steel soil corer “Vienna Scientific Instruments, Vienna, Austria”. In the field, a battery-operated digital weighing balance was used to weigh 50 grams of soil. It was then put in polybags and sealed so that it could be transported to the laboratory. The samples of the soil were dried in the laboratory at 100°C until a constant weight was achieved. The moisture content of the soil was then determined on the dry weight basis [39].

### Water potential

The water potential of both tree twigs and seedlings was measured with a “Pressure chamber (PMS Instrument Co. model 1000) on individually marked separate trees at specific times of the day and seasonal intervals [13,21,22,26]). Trees twig water potential measurement, samples were taken at 1–2 m height and diameter of 1–2 mm, approximately 15 cm long leaf-bearing, then this twig was inserted into the pressure chamber. External pressure from N_2_ gas was applied in the pressure chamber till a severe bubbling was detected at the cut twig end indicating pressure considered as the Balance Pressure (BP). The negative BP value is taken as the water potential (Ψ = -BP). Measurements were conducted at two specific times during the day: pre-dawn (Ψ_PD_) between 5:30−6:30 a.m. and midday (Ψ_MD_) between 1:30−2:30 p.m.

### Water potential components

To create a connection between the components of water potential and RWC, we used Pressure-Volume (PV) curves prepared with the help of a Pressure chamber. The “PV curves” were constructed that followed the “bench-drying method” for both-the seedlings and the trees. At each study site, 20 foliated twigs were collected for creating the PV curves. These twigs were then stored in an insulated plastic container to maintain their freshness. Subsequently, after bringing them to the laboratory, their cut ends were re-cut under water. For re-saturation, the re-cut twigs were then left overnight. Leaving them for 12 hrs, the twigs were subjected to successive measurements of the weight of the twig and the Ψ of the plant while being allowed to dehydrate gradually. This process enabled the researchers to obtain data points for constructing the PV curves, which illustrate the relationship between Ψ and RWC%. From the constructed PV curves, several water potential components were determined. These components included:

➢ Osmotic potential at full turgor (OP_Full_): The water potential at the point of full turgor, represents the maximum hydration state of the plant cells.
➢ Osmotic potential at zero turgor (OP_Zero_): The water potential at the point of zero turgor, represents the point at which the cells lose all water and become completely dehydrated.
➢ Relative Water Content at turgor loss point (RWCz): The RWC% value corresponds to the turgor loss point, which is the point at which the cells lose their turgidity and start to lose water.

Additionally, the reduction in osmotic potential was distinctly premeditated for diverse seasons to observe changes and adjustments in osmotic potential over different periods.

### Conductance of leaf

Measurements of leaf conductance of both the trees and the seedlings were taken seasonally by making use of an AP_4_ leaf-porometer manufactured by Delta-T Devices. The data on conductance was collected at specific times: between 10:30 and 11:30 in the morning (gw_AM_), and between 1:30 and 2:30 in the afternoon. These measurements were directed on five leaves per individual on the sunlit side [13,21,22,26].

### Statistical analysis

The obtained data was analyzed using the ANOVA test. The SPSS software version 2016 was used for these analyses. Sites, seasons, and years of interface were variables of ANOVA. To determine the relationship between variables, the Spearman rank correlation coefficient (r) was calculated.

## Results

### Moisture in soil

**Fig 2:**
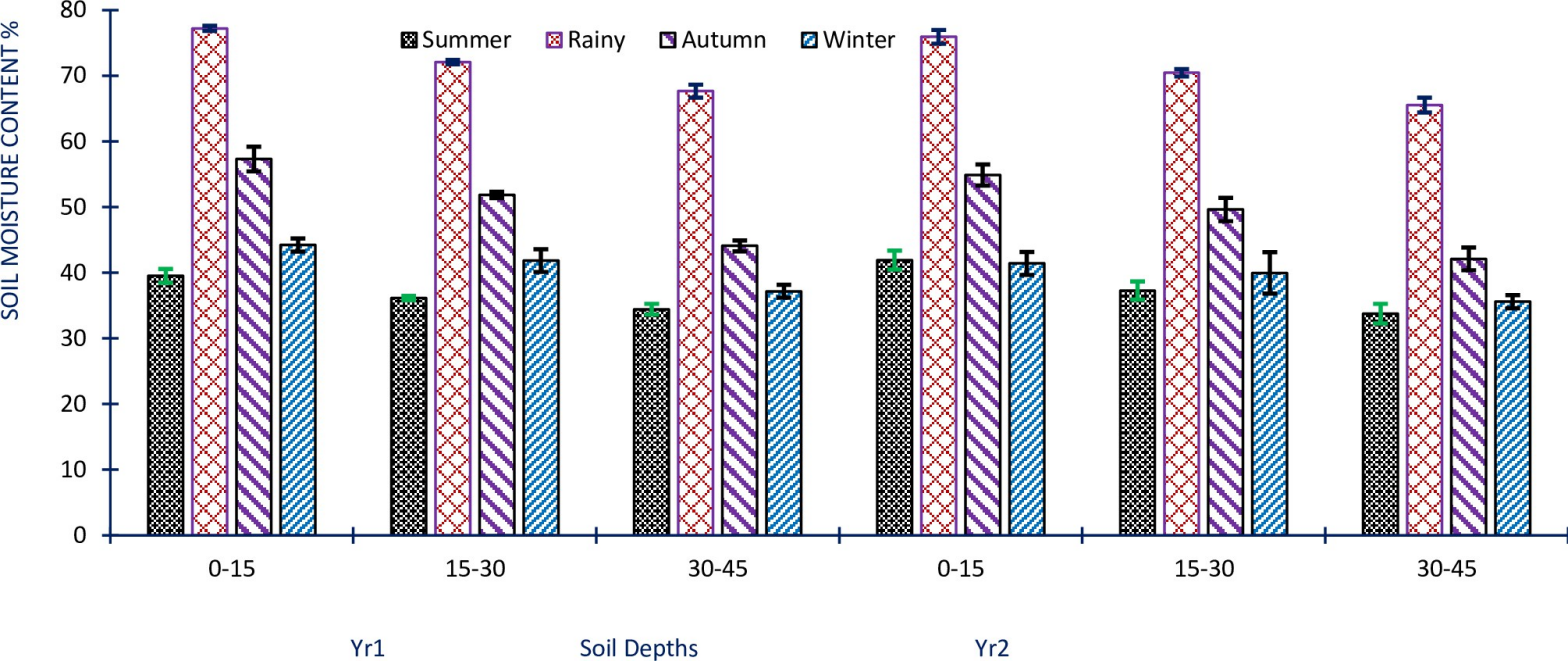
The mean soil moisture content across the studied sites, seasons, and years. The error bars indicate ± SE.

The soil moisture data collected from different depths showed significant variability (p<0.05). The Sm values exhibited fluctuations across all study sites and seasons, with different depths showing varying levels of soil moisture content. In the first year (Yr1), Sm ranged from 34.45±0.32 to 77.22±2.21%, and in the second year (Yr2), it ranged from 33.77±2.72 to 75.93±3.19% across all study sites and seasons. These results indicate that the Sm fluctuated over the two years of the study and exhibited variations across the different sites and seasons. The maximum Sm values were observed at a depth of 0–15 cm during the rainy season. This indicates a higher content of Sm in this period. On the other hand, the minimum Sm values were found at a depth of 30–45 cm during the summer season. This suggests lower Sm content during these dry periods (Fig 2). The statistical analysis using ANOVA confirmed significant variations in Sm levels in the study sites, seasons, and years, with p<0.05.

### Trees water potential

**Fig 3:**
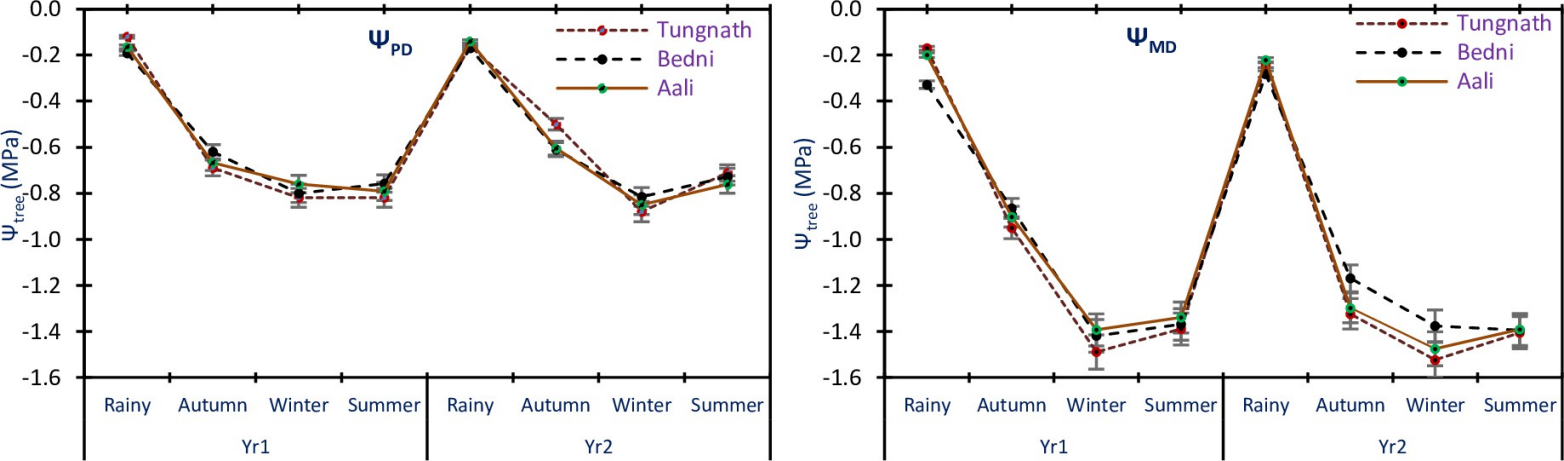
Mean Ψ_PD_ and Ψ_MD_ of *R*. *campanulatum* trees twig in the sites of study and seasons. The error bars indicate ± SE. The different colored lines indicate the three sites.

The Ψ_PD_ values of tree twigs exhibited variations between -0.12±0.03 and -0.82±0.10 MPa in Yr1 and between -0.14±0.14 and -0.88±0.22 MPa in Yr2. Similarly, Ψ_MD_ of trees twigs, the values ranged from -0.17±0.13 to -1.49±0.12 MPa in Yr1 and from -0.22±0.23 to -1.52±0.31 MPa in Yr2, in all the study sites and seasons (Fig 3). The results indicated that the Ψ_PD_ was slightly higher in Yr2 compared to Yr1, but the difference was not statistically significant, as ANOVA did not show any significant variation between the two years for Ψ_PD_. However, for Ψ_MD_, there were significant variations between the two years, as ANOVA demonstrated significant differences across the study sites, years, and seasons, including all interactions between Ψ_PD_ and Ψ_MD_ (p<0.05). In all the sites of study and both the years, the Ψ_PD_ and Ψ_MD_ were most negative in the winter season, indicating increased water stress during this dry period. The least negative values for Ψ_PD_ and Ψ_MD_ were observed during the rainy season, indicating a higher water availability and lower water stress during this period. The ANOVA results revealed significant variations in Ψ_PD_ across the study sites and seasons (p<0.05). However, there were no significant differences between the two years. On the other hand, the Ψ_MD_ showed significant variations across the study sites, years, and seasons, as well as significant interactions between Ψ_PD_ and Ψ_MD_ (p<0.05).

### Seedlings water potential

**Fig 4:**
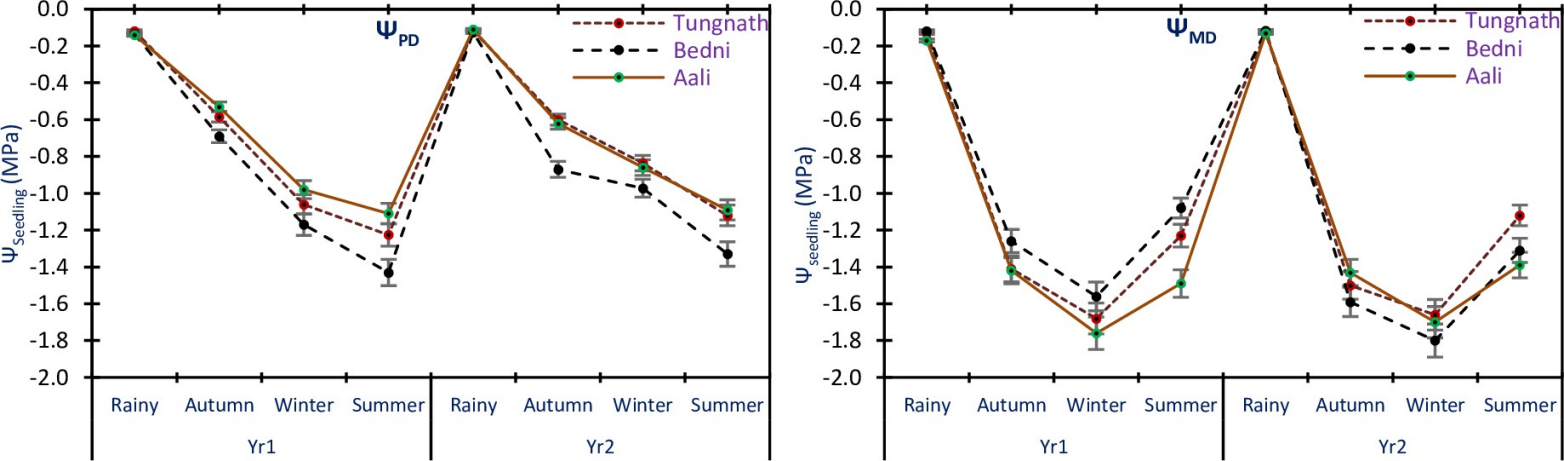
Mean Ψ_PD_ and Ψ_MD_ of *R*. *campanulatum* seedlings in all the sites of study and seasons. The error bars indicate ± SE. The different colored lines reflect the three sites of study.

The Ψ_PD_ values of seedlings showed variations between -0.12±0.05 and -1.43±0.42 MPa in Yr1 and between -0.11±0.06 and -1.33±0.29 MPa in Yr2. For Ψ_MD_, the values were -0.12±0.08–1.76±0.33 MPa in Yr1; -0.12±0.02 to -1.80±0.76 MPa in Yr2, in all the sites of study and seasons (Fig 4). The results indicated that the Ψ_PD_ of seedlings did not differ significantly between the two years of the study, as indicated by the non-significant ANOVA results. However, for Ψ_MD_ of seedlings, there were no significant differences between the two years, except for the significant interaction with the season and species (p<0.05). In all sites of the study and both years, Ψ_PD_ of seedlings was most negative during the summer season, indicating increased water stress during this period. On the other hand, Ψ_MD_ was most negative during the winter season, suggesting that seedlings experienced higher water stress during this time. In contrast, both Ψ_PD_ and Ψ_MD_ was the least negative during the rainy season, indicating a relatively higher water availability and reduced water stress during this period. The ANOVA results revealed significant variations in Ψ_PD_ of seedlings in the years, seasons, and sites of the study (p<0.05). Additionally, significant interactions were observed between Ψ_PD_ and different factors. For Ψ_MD_ of seedlings, significant variations were found in the sites and seasons (p<0.05), with significant relations observed with the season and species (p<0.05), but no noteworthy alterations were noted between the two years of the study.

### Seasonal change in water potential

**Fig 5:**
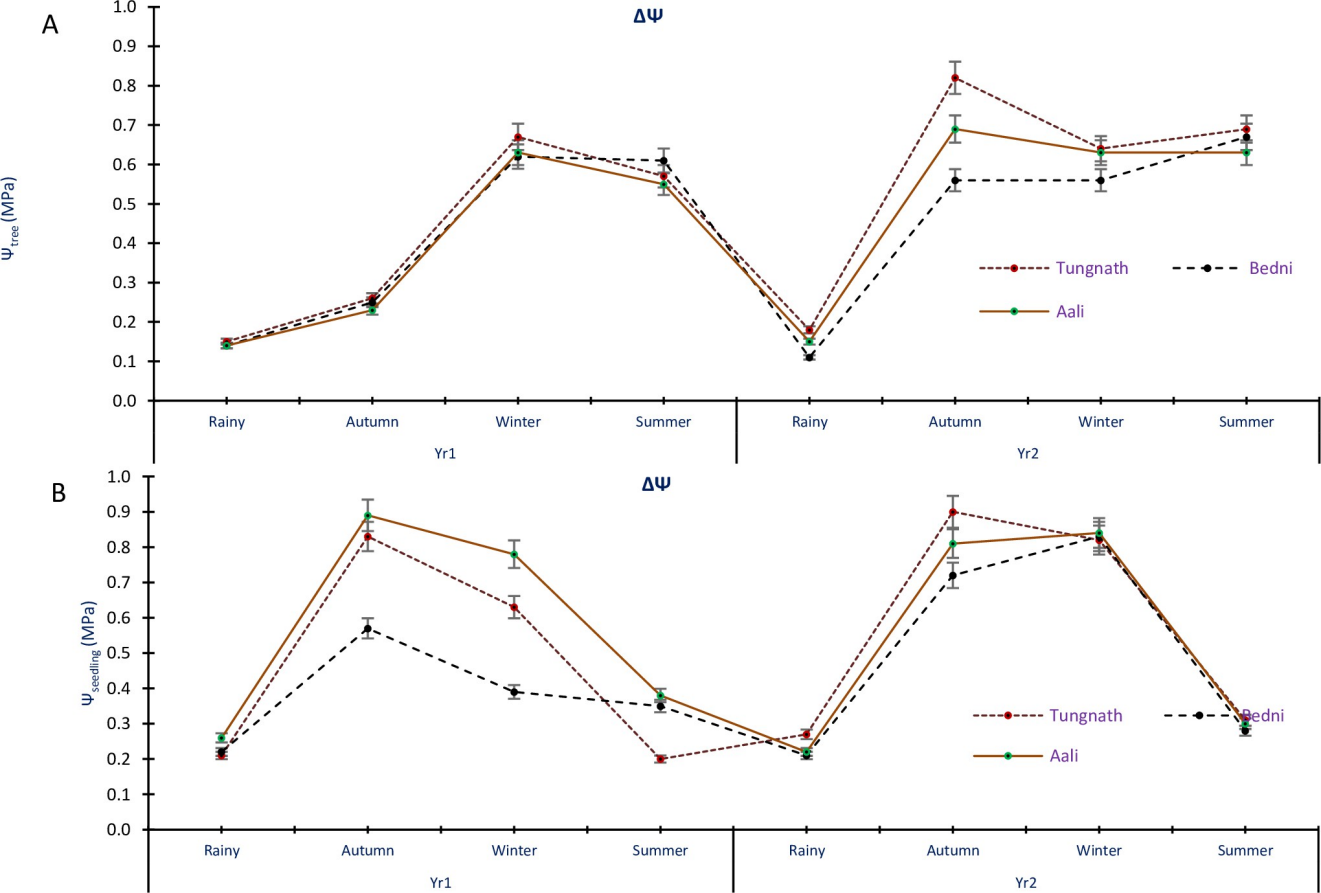
Seasonal variations in water potential in *R*. *campanulatum* trees (A) and seedlings (B) across the seasons and sites. Error bars indicate ± SE. The different colored lines show the three sites.

The mean seasonal change (ΔΨ = Ψ_MD_—Ψ_PD_) in tree twigs water potential exhibited variations, ranging between 0.14±0.08 and 0.67±0.01 MPa in Yr1 and between 0.11±0.05 and 0.82±0.09 MPa in Yr2, in study sites and seasons. For both years, seasonal change in the water potential of the tree twigs was minimal in the rainy season and maximum in the winters in Yr1, in the autumn period in Yr2 (Fig 5). Similarly, the mean ΔΨ in water potential of seedling was 0.20±0.11; 0.89±0.23 MPa in Yr1; 0.21±0.09 and 0.90±0.33 MPa in Yr2, in all study sites and seasons. For both years, the maximum ΔΨ in seedling water potential was observed during the autumn season, while the minimum ΔΨ occurred in the summer period in Yr1, and during the rainy season in Yr2 (Fig 5). ANOVA results indicated that the ΔΨ in both trees and the seedlings speckled pointedly in the years, seasons and the sites of study (p<0.05). This suggests that the seasonal fluctuations in Ψ were influenced by environmental conditions and varied across different locations and times of the year.

### Components of tree water potential

**Fig 6:**
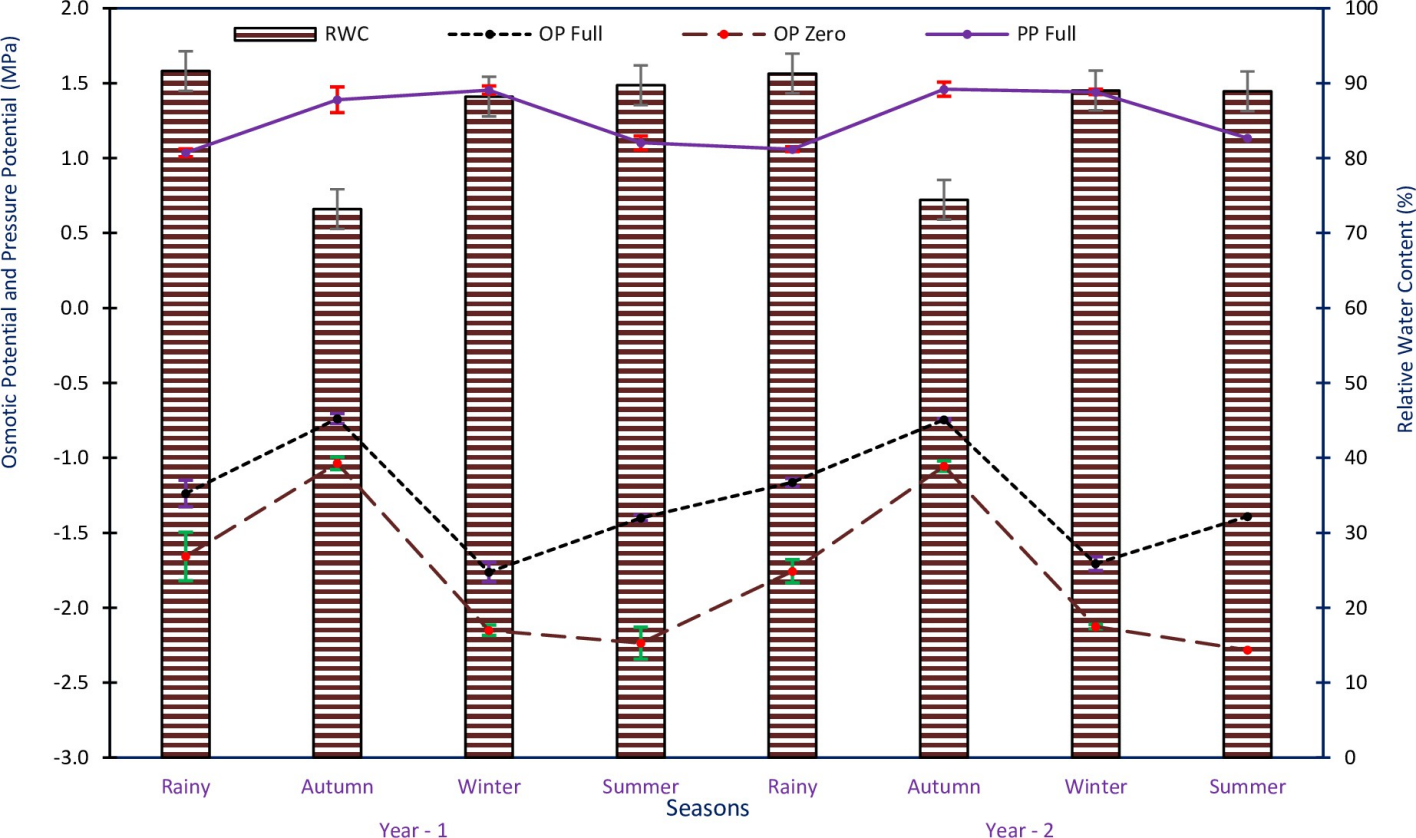
Osmotic potential at (full turgor and at zero turgor); pressure potential at full turgor, and relative water content of *R*. *campanulatum* trees in sites of study and seasons for two years of study period. Error bars indicate ± SE.

The osmotic potential at full turgor exhibited variations, ranging from -0.74±0.03 to -1.76±0.06 MPa in Yr1 and from -0.75±0.01 to -1.71±0.02 MPa in Yr2, in study sites and seasons. Similarly, the osmotic potential at zero turgor ranged from -1.04±0.04 to -2.24±0.11 MPa in Yr1 and from -1.06±0.03 - -2.28±0.09 MPa in Yr2, across study sites and seasons. Both OP_Full_ and OP_Zero_ were least negative in the autumn period, while OP_Full_ was most negative during the winter season and OP_Zero_ in the summer season, for both years (Fig 6). The data showed that OP_Full_ declined from autumn to winter season, with a decrease of -1.02 MPa in Yr1 and -0.96 MPa in Yr2. Similarly, OP_Zero_ declined from autumn to summer season, with a decrease of -1.20 MPa in Yr1 and -1.22 MPa in Yr2. The pressure potential at full turgor (PP_Full_) ranged 1.04±0.03–1.45±0.03 MPa in Yr1; 1.06±0.02–1.46±0.05 MPa in Yr2, in all study sites and seasons. The PP_Full_ was minimum in the rainy period and maximum in the winters in Yr1, and during the autumn season in Yr2 (Fig 6). RWC% of the trees ranged from 73.20±0.61 to 91.62±0.51% in Yr1 and from 74.44±0.46 to 91.30±0.52% in Yr2, across study sites and seasons. RWC% was minimum in the autumn season and maximum in the rainy period in both years (Fig 6). ANOVA results indicated that OP_Full_, OP_Zero_, PP_Full_, and RWC% were wide-ranging meaningfully in all sites of study and seasons (p<0.05). However, there were no significant differences between the two years of the study. The interaction between sites and seasons showed significant variation, but other interactions were non-significant.

### Components of seedling’s water potential

**Fig 7:**
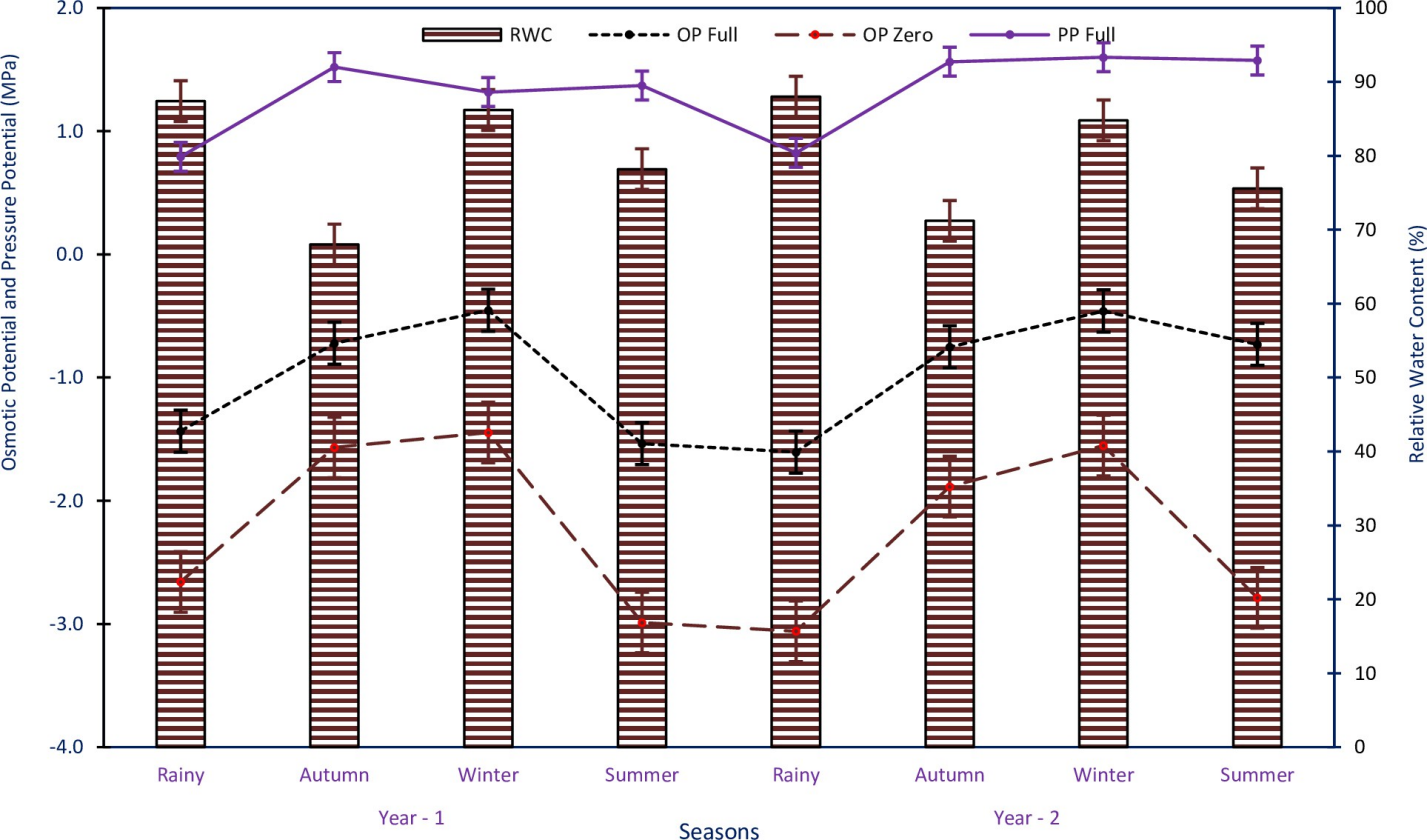
Osmotic potential at (full turgor and at zero turgor); pressure potential at full turgor, and relative water content of *R*. *campanulatum* seedlings in sites and seasons for two years of study period. Error bars indicate ± SE.

The OP_Full_ exhibited variations, ranging from -0.45±0.37 to -1.53±0.37 MPa in Yr1 and from -0.45±0.12 to -1.60±0.12 MPa in Yr2, in study sites and seasons. Similarly, the OP_Zero_ ranged from -1.44±0.28 to -2.98±0.45 MPa in Yr1; and -1.55±0.13 to -3.06±0.30 MPa in Yr2, in sites of study and seasons. Both OP_Full_ and OP_Zero_ were least negative in the winter season and most negative in the summer period in Yr1, and in the rainy season in Yr2 (Fig 7). The data indicated that OP_Full_ and OP_Zero_ decreased from the winter to the summer season in both years. The decrease in OP_Full_ was -1.08 MPa in Yr1 and -0.28 MPa in Yr2. Similarly, OP_Zero_ reduced from -1.44 to -2.98 MPa in Yr1 and -1.55 to -2.78 MPa in Yr2, with a decline of -1.54 MPa in Yr1 and -1.23 MPa in Yr2 (Fig 7). The PP_Full_ varied between 0.79±0.17 and 1.50±0.19 MPa in Yr1 and between 0.82±0.18 and 1.60±0.15 MPa in Yr2, across all study sites and seasons. PP_Full_ was minimum during the rainy period for both years and was maximum in the autumn season for Yr1 and in the winter season for Yr2 (Fig 7). The RWC% of *R*. *campanulatum* seedlings ranged from 68.00±3.17 to 87.40±1.24% in Yr1 and from 71.20±3.73 to 88.00±1.09% in Yr2, across all study sites and seasons. RWC% was lowest during the autumn season and highest during the rainy period in both years (Fig 7). The ANOVA results indicated that OP_Zero_, PP_Full_, and RWC% of *R*. *campanulatum* seedlings speckled significantly in the study sites and seasons (p<0.05), but not amid the two study years. OP_Full_ varied significantly across the study sites (p<0.05), but not between the two study years and seasons. The interface of sites and seasons presented noteworthy disparity (p<0.05), while the other connections were non-significant.

### Conductance of tree leaf

**Fig 8:**
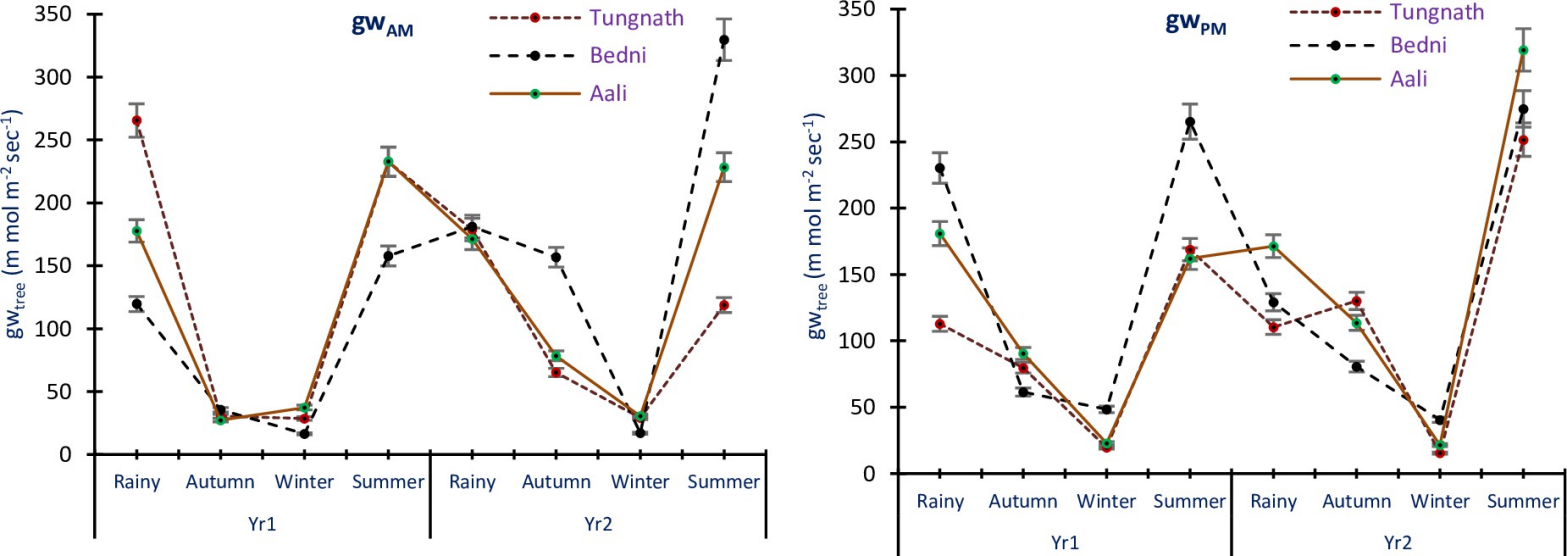
The mean conductance of leaf in morning and afternoon for *R*. *campanulatum* trees in all the seasons, sites, and years. The error bars indicate ± SE. Different colored lines designate the three sites.

The conductance of leaf in the morning (gw_AM_) for *R*. *campanulatum* trees exhibited variations, ranging from 28.59±0.50 to 265.44±9.31 m mol m^-2^ sec^-1^ in Yr1; from 29.15±0.33 to 329.68±3.77 m mol m^-2^ sec^-1^ in Yr2, across all study sites and seasons. Similarly, the afternoon leaf conductance (gw_PM_) ranged from 19.50±1.82 to 265.22±5.70 m mol m^-2^ sec^-1^ in Yr1; from 15.42±0.54 to 319.26±8.30 m mol m^-2^ sec^-1^ in Yr2, in all study sites and seasons. Both gw_AM_ and gw_PM_ were minimum in the winter season and maximum in the summer season, followed by the rainy time (Fig 8). The ANOVA results indicated conductance of leaf in the morning and afternoon for *R*. *campanulatum* trees as wide-ranging significantly with years, sites, and seasons (p<0.05). Additionally, the exchanges of the leaf conductance in the morning were found to be significant (p<0.05).

### Conductance of seedlings leaf

**Fig 9:**
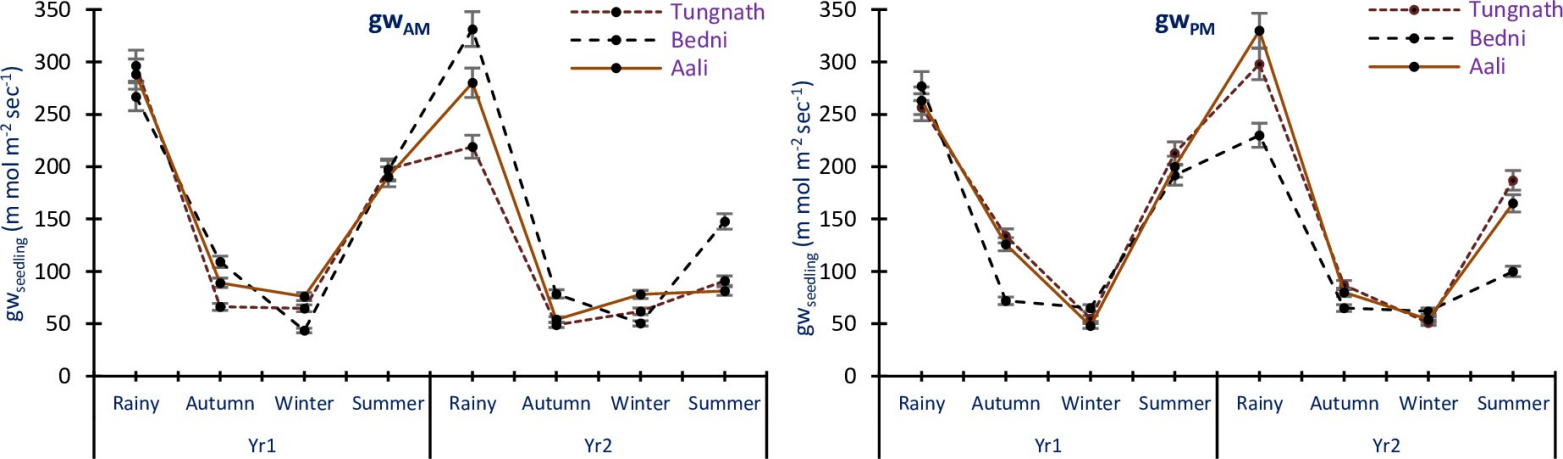
Mean conductance of leaf in morning and afternoon of *R*. *campanulatum* seedlings across all the seasons, sites, and years. Error bars indicate ± SE. Different colored lines specify the three sites.

The gw_AM_ of *R*. *campanulatum* seedlings displayed variations, ranging from 43.60±3.51 to 296.40±12.68 m mol m^-2^ sec^-1^ in Yr1 and from 49.00±3.38 to 331.40±8.94 m mol m^-2^ sec^-1^ in Yr2, across all study sites and seasons. Similarly, the gw_PM_ ranged from 48.00±3.33 to 277.00±19.20 m mol m^-2^ sec^-1^ in Yr1 and from 51.00±2.04 to 330.00±2.67 m mol m^-2^ sec^-1^ in Yr2, in study sites and seasons. Both gw_AM_ and gw_PM_ were highest in the rainy season and lowest during the winter and autumn seasons (Fig 9). ANOVA results indicated the conductance in the morning and afternoon for *R*. *campanulatum* seedlings as diverse significantly with study years, seasons, and sites (p<0.05). Additionally, all the exchanges were also found to be significant (p<0.05).

### Relationships amongst variables

**Fig 10:**
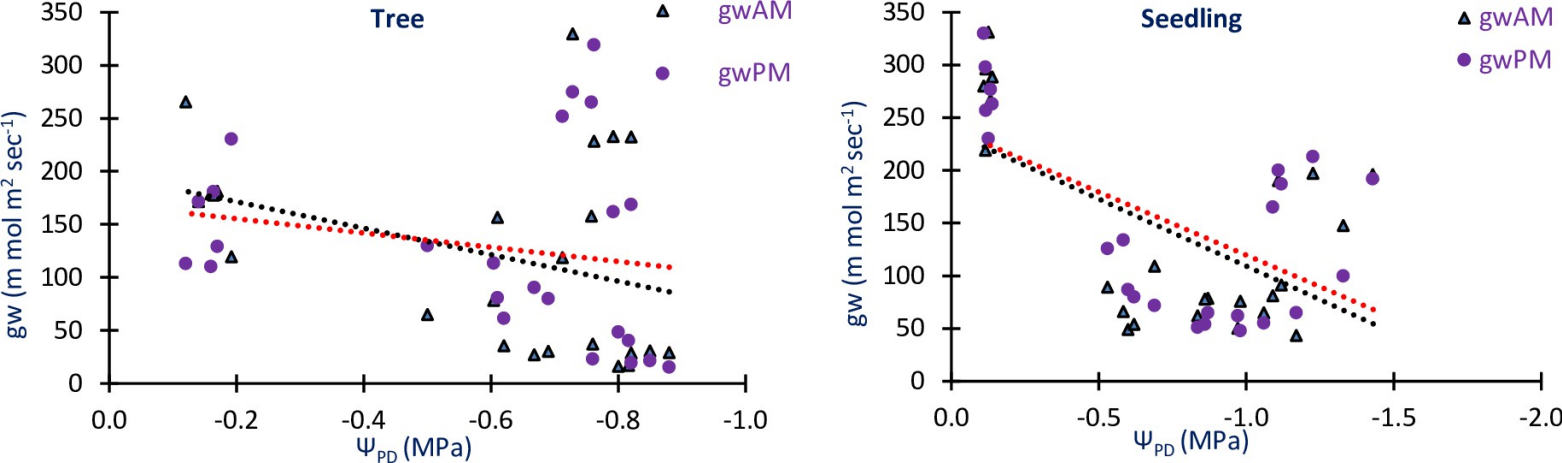
Relationships of conductance of leaf in the morning and afternoon with pre-dawn water potential of *R*. *campanulatum* trees and seedlings.

The analysis revealed that the soil moisture levels at depths of Sm_15_, Sm_30_, and Sm_45_ were not significantly correlated with the Ψ_PD_ of both trees and seedlings. Additionally, Sm_45_ was not significantly correlated with the Ψ_MD_ of trees and seedlings. However, all other variables showed significant correlations with each other at both the 0.01 and 0.05 implication levels (p<0.01 and 0.05) (Table 1). Regarding the conductance of leaves in the morning and afternoon for *R*. *campanulatum* trees, the highest values were observed when the Ψ_PD_ was between -0.6 to -0.8 MPa. Similarly, in *R*. *campanulatum* seedlings, the maximum morning and afternoon leaf conductance occurred when the Ψ_PD_ ranged from -0.5 to -1.0 MPa (Fig 10).

**Table 1:** Spearman rank correlation coefficients amid diverse water relations characteristics of the study species.

Variable	Sm_15_	Sm_30_	Sm_45_	Tree				Seedling			
				Ψ_PD_	Ψ_MD_	gw_AM_	gw_PM_	Ψ_PD_	Ψ_MD_	gw_AM_	gw_PM_
**Sm**_**15**_	1	0.96**	0.84**	-0.28^NS^	-0.31**	-0.60**	-0.49**	-0.07^NS^	-0.53**	-0.61**	-0.62**
**Sm**_**30**_		1	0.95**	-0.21^NS^	-0.25**	-0.54**	-0.39**	-0.03^NS^	-0.46*	-0.56**	-0.54**
**Sm**_**45**_			1	-0.08^NS^	-0.11^NS^	-0.50**	-0.36**	0.09^NS^	-0.33^NS^	-0.47*	-0.44*
**Ψ**_**PD**_				1	0.95**	0.37**	0.20**	1	0.76**	0.58**	0.57**
**Ψ**_**MD**_					1	0.31**	0.13**		1	0.91**	0.90**
**gw**_**AM**_						1	0.71**			1	0.87**
**gw**_**PM**_							1				1

NS = P>0.05

** = 0.05>P>0.01

* = 0.01>P>0.001.

## Discussion

Extreme water potentials can lead to extensive xylem cavitation, resulting in foliage dieback or even plant death [40]. At high-altitude regions of the Himalayas, the tree-water relation is influenced by the unique environmental conditions. Trees growing at high altitudes face several challenges related to water availability, temperature, and atmospheric conditions. At high elevations, an increase in water vapor may reduce the significance of solar brightening while increasing the importance of low temperatures, as water availability to plants rises [41].

Trees at high altitudes often rely on precipitation, such as snow and rain, as their primary water source. In the Himalayas, snowfall is common, and the melting snow during spring and summer provides an essential water supply for plants. The availability of water can vary with elevation, seasonal changes, and monsoon patterns. During the study, Sm was observed to be high but decreased with increasing soil depths across all treeline sites. The moisture content of the soil directly affects the water relation of *R*. *campanulatum* trees and seedlings. In areas with high rainfall or proximity to water sources, the soil moisture might remain relatively high, promoting the development and existence of the species. The abundant moisture could be attributed to the high rainfall and the presence of the krummholz community, which thrives in moist soils.

Throughout the study sites, various seasons, and two study years, the Ψ of *R*. *campanulatum* trees and seedlings did not remain above critical levels (-0.88 and -1.43 MPa for trees and seedlings, respectively), indicating the absence of severe stress for both. The snowmelt from snowfall in winter maintains soil moisture during spring and summer, before the monsoon rains [13], which plays a significant role in mitigating severe stress in Himalayan treeline species. A reduction in snowpack due to climate warming would significantly affect the water relations of *R*. *campanulatum* by decreasing the availability of soil moisture during critical spring and summer growth periods. This could exacerbate water stress, leading to more negative water potentials, ultimately compromising the species’ survival and growth reported on *Abies spectabilis* and *Quercus semecarpifolia* in the high-altitude region of Himalaya [21,22]. Similar studies reported that both *R*. *arboreum* and *R*. *campanulatum* trees had Ψ above -1.0 MPa [4] and for *R*. *campanulatum* in comparable treeline areas [13]. The root system of *Rhododendrons*, including *R*. *campanulatum*, is shallow and fibrous, which enables effective water absorption from the upper soil layers.

Due to small vessel diameters, *Rhododendron* species are sensitive to drought-induced embolism [42]. *R*. *campanulatum* is likely to have some level of drought tolerance, given its adaptation to high-altitude environments with varying water availability. However, it is essential to note that the extent of drought tolerance can vary between individual plants and populations. Consequently, the Ψ of trees and seedlings was most negative during winter seasons when osmotic substances in plant tissues contribute to low Ψ values [40]. Dearth at changed periods of the year leads to diverse retorts and modifications [16]. Across all sites, seasons, and years, the seedlings of *R*. *campanulatum* experienced higher stress levels than the larger individuals. This is primarily due to their reliance on water from shallow layers of soil, where soil dampness obtain ability depletes speedily [43], also supports this notion, noting that small tree seedlings have more negative Ψ values and are more severely stressed compared to larger individuals at a particular site [22,26].

Monitoring the seasonal changes in water status is a crucial tool for understanding the overall water relations of a tree. In our study, we observed distinct differences in seasonal change patterns from 0.20 to 0.90 MPa. These variations were most pronounced during the autumn season and least during the rainy seasons. Previous studies reported ΔΨ values between 0.06 and 0.90 MPa for *Pinus roxburghii*, *R*. *arboreum*, and *Quercus leucotrichophora* [16], similarity values were recorded from 1.07 to 1.22 MPa for treeline species [13]. Regular fluctuations in the Ψ of plants are a result of the combined effects of environmental factors and plant physiology [21,26]. Higher ΔΨ values were normally related to elevated transpiration rates, negative leaf water potential, and increased photosynthetic activity. Treeline species exhibited lower ΔΨ because of precise climatic circumstances, such as precipitation, temperature, soil dampness, plant composition, and moisture. Interestingly, the ΔΨ usually emaciated when Ψ_PD_ was above -0.5 MPa, indicating a healthier water status, and tended to decline with increasing stress levels. Understanding these seasonal changes in Ψ helps us comprehend how trees respond to environmental conditions and water availability in their habitats. Projected climate changes, including altered monsoon patterns and rising temperatures, may heighten water stress for *R*. *campanulatum* by decreasing snowmelt and shifting precipitation. This could compel the species to depend more on osmotic adjustment and stomatal regulation, potentially hindering growth and reproductive success. Over time, such stresses may push *R*. *campanulatum* and other treeline species to migrate upward, contributing to the densification and upward shift of treelines in the Himalayas.

The decline in osmotic potential is documented to cope with scarcity and salinity stress [44]. In our study, we observed differences in the degree of osmotic decline between *R*. *campanulatum* trees and seedlings both at zero and full turgor, with seedlings showing higher levels of stress compared to trees. The decrease in osmotic potential in trees occurred from autumn to summer at zero turgor and from autumn to winter at full turgor. On the other hand, seedlings showed a decline from winter to summer at both zero and full turgor. Similar results were reported in some other treeline species [13]. The pronounced lowering of osmotic potential indicates the approach is to safeguard water accessibility, particularly during warm and sunny days with optimal humidity, when soil is not knowingly dry, and leaves are completely developed. Osmotic adjustment takes place when OP_Full_ drops [45]. In our study, the species exhibited seasonal deviations in OP_Full_, ranging from -0.74 to -1.76 MPa in trees and -0.45 to -1.53 MPa in seedlings. A wider range of osmotic potential values is described at complete and nil turgor across seasons for Himalayan tree species [16], with the lowest osmotic potential values being additionally negative. This pronounced osmotic potential decline during mild drought conditions ensures that soil water is accessible to continue photosynthesis. The relative water content at zero turgor (RWCz), which indirectly measures a plant’s ability to maintain its turgor under declining Ψ, was high in *R*. *campanulatum*, ranging from 75.60 to 89.73% in both trees and seedlings during winter and summer seasons. The power to continue high RWC under deficiency was also detected [13,46,47]. The observed osmotic adjustments and high RWC values demonstrate how *R*. *campanulatum* trees and seedlings adapt to maintain water availability and turgor during various seasons and drought conditions. These adaptations are crucial for their survival and photosynthetic activity in the challenging high-altitude environments of the Himalayas.

The water relations of *R*. *campanulatum* in the Himalayas exhibit physiological adaptations similar to those observed in treeline species in other mountain ranges. In continental alpine timberlines, an incomplete development of cuticles during the short growing period seems to play the most important role in determining severe drought conditions in the winter [17,48]. In arctic, temperate-maritime, and tropical treelines, cold temperatures have a major impact on limiting physiological processes [49]. *R*. *campanulatum*, species in these regions rely on osmotic adjustment, high relative water content, and stomatal regulation to cope with water stress caused by fluctuating seasonal moisture availability and temperature extremes [4]. The physiological responses to water stress among treeline species are broadly consistent globally, though local climatic conditions introduce variations in the degree and timing of these adaptations.

Transpiration is the process by which plants lose water vapor through small pores called stomata on their leaves. *R*. *campanulatum*, being an evergreen species, undergoes transpiration throughout the year. This process is vital for nutrient transport and cooling of the plant but can lead to water loss if not balanced with water uptake from the roots. All evergreen species with broad leaves maintained open stomata all over the year, but their leaf conductance exhibited varying degrees of decline in response to hostile climatic conditions [13]. In our study, across various sites, seasons, and two study years, the gw_AM_ and gw_PM_ values of *R*. *campanulatum* trees ranged from 28.59 to 329.68 and 15.42 to 319.26 m mol m^-2^ sec^-1^, respectively. For seedlings, the values were 43.60 to 331.40 m mol m^-2^ sec^-1^ in the morning and 48.00 to 330.00 m mol m^-2^ sec^-1^ in the afternoon. The gw_AM_ values of 27.26 and 274.82 m mol m^-2^ sec^-1^ were reported for *R*. *campanulatum* [4], and values of 28.1 and 289.5 m mol m^-2^ sec^-1^ were found in similar treeline areas [13]. Extreme leaf conductance in *R*. *campanulatum* occurred during the rainy and summer seasons. Conversely, leaf conductance was lowest during dry months for both trees and seedlings. During dry months, when tree water potential reached its lowest, stomatal conductance decreased [30]. Seasonal variations in leaf conductance, driven by stomatal behavior, play a critical role in photosynthesis by regulating gas exchange. Higher water availability leads to increased stomatal conductance, enhancing CO_2_ uptake and promoting growth. Conversely, in drier conditions, reduced stomatal conductance limits photosynthesis, potentially stunting growth and affecting overall plant health. Similarly, a reduction in gw in dry periods is a reciprocal function of the leaf water potential (Ψ_PD_) in oak trees [50]. These findings suggest that *R*. *campanulatum* and other broad-leaved evergreen species exhibit specific strategies to cope with changes in water availability and environmental conditions. By adjusting their stomatal conductance, these plants can regulate their water loss and adapt to adverse climatic conditions, which is crucial for their survival and performance in their habitat. The study from 2017 to 2020 found year-to-year variability in physiological parameters like water potential and leaf conductance, but no significant long-term trends in the water relations of R. *campanulatum*. Fluctuations were attributed to seasonal and site-specific conditions rather than a progressive increase in physiological stress, indicating stable water relations despite annual climatic variations.

## Conclusion

The study sheds light on the intricate dynamics of water relations in *R*. *campanulatum* trees and seedlings within the challenging high-altitude environments of the Himalayas. We found that these species face several challenges related to water availability, temperature fluctuations, and atmospheric conditions, which significantly influence their physiological responses. Our findings highlight the species exhibited specific strategies, such as adjusting stomatal conductance, to regulate water loss and adapt to varying water availability throughout the year. Overall, understanding the seasonal changes in water status and physiological responses is essential for comprehending how *R*. *campanulatum* and similar species respond to environmental challenges in their habitat. These insights contribute to our broader understanding of plant-water interactions in high-altitude ecosystems and have implications for the conservation and management of mountainous regions facing climate change-induced water stress.

## Supporting information

S1 File (XLSX)
